# Interareal coupling reduces encoding variability in multi-area models of spatial working memory

**DOI:** 10.3389/fncom.2013.00082

**Published:** 2013-07-01

**Authors:** Zachary P. Kilpatrick

**Affiliations:** Department of Mathematics, University of HoustonHouston, TX, USA

**Keywords:** neural field, bump attractor, spatial working memory, correlations, noise cancelation

## Abstract

Persistent activity observed during delayed-response tasks for spatial working memory (Funahashi et al., 1989) has commonly been modeled by recurrent networks whose dynamics is described as a *bump attractor* (Compte et al., 2000). We examine the effects of interareal architecture on the dynamics of bump attractors in stochastic neural fields. Lateral inhibitory synaptic structure in each area sustains stationary bumps in the absence of noise. Introducing noise causes bumps in individual areas to wander as a Brownian walk. However, coupling multiple areas together can help reduce the variability of the bump's position in each area. To examine this quantitatively, we approximate the position of the bump in each area using a small noise expansion that also assumes weak amplitude interareal projections. Our asymptotic results show the motion of the bumps in each area can be approximated as a multivariate Ornstein–Uhlenbeck process. This shows reciprocal coupling between areas can always reduce variability, if sufficiently strong, even if one area contains much more noise than the other. However, when noise is correlated between areas, the variability-reducing effect of interareal coupling is diminished. Our results suggest that distributing spatial working memory representations across multiple, reciprocally-coupled brain areas can lead to noise cancelation that ultimately improves encoding.

## Introduction

Persistent spiking activity has been experimentally observed in prefrontal cortex (Funahashi et al., 1989; Miller et al., 1996), parietal cortex (Colby et al., 1996; Pesaran et al., 2002), superior colliculus (Basso and Wurtz, 1997), caudate nucleus (Hikosaka et al., 1989; Levy et al., 1997), and globus pallidus (Mushiake and Strick, 1995; McNab and Klingberg, 2008) during the retention interval of visuospatial working memory tasks. Often, the subject must remember a cue's location for several seconds (Funahashi et al., 1989). Delay period neurons persistently fire in response to a preferred cue orientation as described by a bell-shaped tuning curve. Networks of these neurons, with recurrent excitation between similarly tuned neurons and broadly tuned feedback inhibition, can generate spatially localized “bumps.” The position of these bumps encodes the remembered location of the cue (Compte et al., 2000).

Dynamic variability can degrade the accuracy of working memory over time though. Fluctuations in membrane voltage and synaptic conductance can lead to spontaneous spike or failure events at the edge of the bump, causing the bump to wander diffusively (Compte et al., 2000; Laing and Chow, 2001). Bump attractor networks are particularly prone to such diffusive error because bump positions lie on a line attractor where each location is neutrally stable (Amari, 1977). Interestingly, psychophysical data demonstrates spatial working memory error does scale linearly with delay time, suggesting the underlying process that degrades memory is diffusive (White et al., 1994; Ploner et al., 1998). Much theoretical work has examined network properties that might limit memory degradation. Several computational studies have explored networks built from bistable neuronal units, which sustain persistent states that are less susceptible to noise (Camperi and Wang, 1998; Koulakov et al., 2002; Goldman et al., 2003). In addition, synaptic facilitation has been shown to slow the drift of bump position due to internal variability (Itskov et al., 2011). Synaptic plasticity has also be shown to reduce diffusion of bumps in (Hansel and Mato, 2013). Finally, spatially heterogeneous recurrent excitation can reduce wandering of bumps quantizing the line attractor by stabilizing a finite set of bump locations (Kilpatrick and Ermentrout, 2013; Kilpatrick et al., 2013).

Complementary to these possibilities, we propose that interareal coupling across multiple areas of cortex may reduce error in working memory recall generated by dynamic fluctuations. Multiple representations of spatial working memory have been identified in different cortical areas (Colby et al., 1996). This distributed representation makes working memory information readily available for motor (Owen et al., 1996) and decision-making (Curtis and Lee, 2010) tasks. In addition, this redundancy may serve to reduce degrading effects of noise. It is known that several areas involved in oculomotor delayed response tasks are reciprocally coupled to one another (Constantinidis and Wang, 2004; Curtis, 2006). We presume the representation of a spatial working memory in a single area takes the form of a bump in a recurrently coupled neural field. Projections between areas share information about bump position across the multi-area network. Recently, (Folias and Ermentrout, 2011) showed several novel activity patterns emerge when considering neural fields with multiple areas. In addition, recent analyses of spatiotemporal dynamics of perceptual rivalry have exploited dual population neural field models, where activity in each area represents a single percept (Kilpatrick and Bressloff, 2010; Bressloff and Webber, 2012b). In this study, we focus on activity patterns where bumps in each area have positions that remain close.

Our study mostly focuses on a dual area model of spatial working memory, where each area provides a replicate representation of the presented cue. We begin by demonstrating the neutral stability of the bump position in each area, in the absence of noise and interareal projections. Upon including noise and interareal projections, we use a small-noise expansion to derive an effective stochastic differential equation for the position of the bump in each area. The effective system is a multivariate Ornstein–Uhlenbeck process, which we can analyze using diagonalization. The variance of this stochastic process decreases as the strength of connections between areas increases. Variance reduction relies on cancelations arising due to averaging noise between both areas. Thus, when noise is strongly correlated between areas, the effect of interareal coupling is negligible. Lastly, we show this analysis extends to the case of *N* (more than two) areas and that for sufficiently strong interareal connections, variance scales as 1/*N*.

## Materials and methods

### Dual area model of spatial working memory

We consider a recurrently coupled model commonly used for spatial working memory (Camperi and Wang, 1998; Ermentrout, 1998) and visual processing (Ben-Yishai et al., 1995). GABAergic inhibition (Gupta et al., 2000) typically acts faster than excitatory NMDAR kinetics (Clements et al., 1992), and we assume excitatory synapses contain a mixture of AMPA and NMDA components. Thus, we make the assumption that inhibition is slaved to excitation as in (Amari, 1977). We can then describe average activity *u*_1_(*x, t*) and *u*_2_(*x, t*) of neurons in either area by the system (Ben-Yishai et al., 1995; Folias and Ermentrout, 2011; Kilpatrick and Ermentrout, 2013)

(1a) $$\tau \text{d}u_{1}(x,t)=\left[-u_{1}+w_{11}*f(u_{1})+\varepsilon^{1/2}w_{12}*f(u_{2})\right]\text{d}t+\varepsilon^{1/2}\text{d}W_{1}(x,t),$$

(1b) $$\tau \text{d}u_{2}(x,t)=\left[-u_{2}+w_{22}*f(u_{2})+\varepsilon^{1/2}w_{21}*f(u_{1})\right]\text{d}t\;+\;\varepsilon^{1/2}\text{d}W_{2}(x,t),$$

where the effects of synaptic architecture are described by the convolution

(2) $$w_{jk}*f(u_{k})=\int_{-\pi }^{\pi }w_{jk}(x-y)f(u_{k}(y,t))\text{dy},$$

for *j*, *k* = 1, 2, so the case *j* = *k* describes recurrent synaptic connections within a area and *j* ≠ *k* describes synaptic connections between areas (interareal). Several fMRI and electrode recordings have revealed correlations between activity in multiple cortical areas during spatial working memory tasks (Constantinidis and Wang, 2004; Curtis, 2006), such as parietal and prefrontal cortex (Chafee and Goldman-Rakic, 1998). However, it seems the strength of these correlations is often not on the order of the activity itself (di Pellegrino and Wise, 1993). For this reason, we presume the strength of interareal connections is weak 0 ≤ ε^1/2^ « 1. Note, we could choose to make them a different magnitude than the noise, but for analytical convenience, we choose interareal connection and noise magnitude to be roughly the same. Analysis could still be performed in other cases, but it would simply be more complicated. By setting τ = 1, we can assume that time evolves on units of the excitatory synaptic time constant, which we presume to be roughly 10 ms (Häusser and Roth, 1997). The function *w*_*jk*_(*x* − *y*) describes the strength (amplitude of *w*_*jk*_) and net polarity (sign of *w*_*jk*_) of synaptic interactions from neurons with stimulus preference *y* to those with preference *x*. Following previous studies, we presume the modulation of the recurrent synaptic strength is given by the cosine

(3) $$w_{jj}(x-y)=w(x-y)=\cos(x-y),\;j=1,2,$$

so neurons with similar orientation preference excite one another and those with dissimilar orientation preference disynaptically inhibit one another (Ben-Yishai et al., 1995; Ferster and Miller, 2000). Lateral inhibitory type network architectures are supported by anatomical studies of the delay period neurons in prefrontal cortex (Goldman-Rakic, 1995). Our general analysis will apply to any even symmetric function of the distance *x* − *y*, but we typically compute things using (Equation 3) since it eases calculations. Finally, synaptic connections from area *k* to *j* are specified by the weight function *w*_*jk*_(*x* − *y*), and we typically take this to be the function

(4) $$w_{jk}(x-y)=E_{j}+M_{j}\cos(x-y),\;k\neq j$$

where *E*_*j*_ and *M*_*j*_ specify the strength of baseline excitation and modulation projecting to the *j*th area.

Output firing rates are given by taking the gain function *f*(*u*) of the synaptic input, which we usually proscribe to be (Wilson and Cowan, 1973)

$$f(u)=\frac{1}{1+{\text{e}}^{-\gamma (u-\theta )}},$$

and often take the high gain limit γ → ∞ for analytical convenience, so (Amari, 1977)

(5) $$f(u)=H(u-\theta )=\{\begin{array}{ll} 0 & :u<\theta ,\\ 1 & :u\geq \theta . \end{array}$$

Effects of noise are described by the small amplitude (0 ≤ ε « 1) stochastic processes ε^1/2^*W*_*j*_ (*x, t*) that are white in time and correlated in space so that 〈*dW_j_(x, t)*〉 = 0 and

$$\begin{array}{l} \langle \text{d}W_{j}(x,t)\text{d}W_{j}(y,s)\rangle =C_{j}(x-y)\delta (t-s)\text{d}t\text{d}s,\\ \langle \text{d}W_{j}(x,t)\text{d}W_{k}(y,s)\rangle =C_{c}(x-y)\delta (t-s)\text{d}t\text{d}s, \end{array}$$

describing both local and shared noise in either area, *j* = 1, 2 with *j* ≠ *k*. For simplicity, we assume the local spatial correlations have a cosine profile *C*_*j*_(*x*) = *c*_*j*_ cos(*x*). We also typically assume the correlated noise component has cosine profile so *C*_*c*_(*x*) = *c*_*c*_ cos(*x*). Therefore, in the limit *c*_*c*_ → 0, there are no interareal noise correlations, and in the limit *c*_*c*_ → min (*c*_1_, *c*_2_), noise in each area is maximally correlated. For instance, when *c*_1_ = *c*_2_ = *c*_*c*_ = 1, noise in each area is drawn from the same process.

### Multiple-area model of spatial working memory

To incorporate the effects of many coupled, redundant areas encoding a spatial working memory, we consider a model with *N* areas and arbitrary synaptic architecture, given by

(6) $$\tau \text{d}u_{j}(x,t)=\left[-u_{j}+\varepsilon^{1/2}\sum_{k=1}^{N}w_{jk}*f(u_{k})\right]\text{d}t\;+\;\varepsilon^{1/2}\text{d}W_{j}(x,t)$$

where *u*_*j*_ represents neural activity in the *j*th area where *j* = 1, …, *N*. As before, we set τ = 1, so each time unit corresponds to the roughly 10 ms timescale of excitatory synaptic conductance. The weight function *w*_*jk*_(*x* − *y*) represents the connection from neurons in area *k* with cue preference *y* to neurons in area *j* with cue preference *x* as described by (Equation 2). For comparison with numerical simulations, we take weight functions to be the cosines (Equation 3) and (Equation 4) and the firing rate function to be Heaviside (Equation 5). As in the dual area model, noises *W*_*j*_(*x, t*) are white in time and correlated in space so that 〈*dW_j_(x, t)*〉 = 0 and

$$\langle {\text{dW}}_{\text{j}}(x,t){\text{dW}}_{\text{k}}(y,s)\rangle =C_{jk}(x-y)\delta (t-s)\text{dtds},$$

with *j, k* = 1,…, *N*, where local noise correlations are described when *j* = *k* and noise correlations between areas are described when *j* ≠ *k*. For comparison with numerical simulations, we consider *C*_*jj*_ (*x*) = cos(*x*) and *C*_*jk*_ (*x*) = *c*_*c*_ cos(*x*) for all *j* ≠ *k*.

### Numerical simulation of stochastic differential equations

The spatially extended model (Equation 1) was simulated using an Euler–Maruyama method with a timestep 10^−4^, using Riemann integration on the convolution term with 2000 spatial grid points. To compute and compare the variances 〈Δ_1_(*t*)^2^〉 for the dual and multiple area model, we simulated the system 5000 times. The position of the bump Δ_*j*_ at each timestep, in each simulation, was determined by the position *x* in each area *j* at which the maximal value of *u*_*j*_(*x, t*) was attained. The variance was then computed at each timepoint and compared to our asymptotic calculations.

## Results

We will now study how interareal architecture affect the dynamics of bumps in multiple area stochastic neural fields. To start, we demonstrate that in the absence of reciprocal connectivity between areas bump attractors exist that are neutrally stable to perturbations that change their position, which has long been known (Amari, 1977; Camperi and Wang, 1998; Ermentrout, 1998). Introducing weak interareal connectivity can decrease the variability in bump position because noise that moves bumps in the opposite direction is canceled due to an attractive force introduced by connectivity. Perturbations that push bumps in the same direction are still integrated, so bumps wander due to dynamic fluctuations, but their effective variance is smaller than it would be without interareal synaptic connections. In the presence of noise correlations between areas, effects of noise cancelation are weaker since stochastic forcing in each area is increasingly similar. Our asymptotic analysis is able to explain all of this with its resulting multivariate Ornstein–Uhlenbeck process.

### Bumps in the noise-free system

To begin, we seek stationary solutions to Equation (1) in the absence interareal connections and noise (ε → 0). Similar analyses have been carried out for bumps in single area populations (Ermentrout, 1998; Hansel and Sompolinsky, 1998). For this study, we assume recurrent connections are identical in all areas (*w*_*jj*_ = *w*). Relaxing this assumption slightly does not dramatically alter our results. Note first stationary solutions take the form (*u*_1_(*x, t*), *u*_2_(*x, t*)) = (*U*_1_(*x*), *U*_2_(*x*)). In the absence of any interareal connections, we would not necessarily expect the peaks of these bumps to be at the same location. However, translation invariance of the system (Equation 1) allows us to set the center of both bumps to be *x* = 0 to ease calculations. The stationary bump solutions then satisfy the system

(7) $$U_{1}=w*f(U_{1}),\;U_{2}=w*f(U_{2}),$$

so the shape of each bump is only determined by the local connections *w*. For *w* given by Equation (3), since *U*_1_(*x*) and *U*_2_(*x*) are assumed to be peaked at *x* = 0, then by also assuming even symmetric solutions, we find

(8) $$\begin{array}{l} U_{1}(x)=\int_{-\pi }^{\pi }\cos yf(U_{1}(y))\text{dy}\cos\text{x},\\ U_{2}(x)=\int_{-\pi }^{\pi }\cos yf(U_{2}(y))\text{dy}\cos\text{x}, \end{array}$$

where we use cos(*x* − *y*) = cos*x* cos*y* + sin*x* sin*y*. We can more easily compute the precise shape of these bumps in case of a Heaviside firing rate function (Equation 5). There is then an identical active region of each bump such that *U*_1_(*x*) > θ and *U*_2_(*x*)> θ when *x* ∈ (−*a*, *a*), so the Equation (8) become *U*_1_(*x*) = U_2_(*x*) = 2sin*a* cos*x*. Applying self-consistency, *U*_1_(±*a*) = *U*_2_(±*a*) = θ, we can generate an implicit equation for the half-widths of the bumps *a* given by 2sin*a* cos*a* = sin(2*a*) = θ. Solving this explicitly for *a*, we find two solutions on $a\in [0,\pi ]:a_{u}=\frac{1}{2}\sin^{-1}\theta$ and $a_{s}=\frac{\pi }{2}-\frac{1}{2}\sin^{-1}\theta$. Only the bump associated with *a*_*s*_ is stable.

The bumps (Equation 7) are neutrally stable to perturbations in both directions, which can lead to encoding error once the effects of dynamic fluctuations are considered (Kilpatrick et al., 2013). Since the two areas are uncoupled, examining bumps' stability can be reduced to studying each bump's stability individually (see Kilpatrick and Ermentrout, 2013 for details). Translating a bump by a scaling of the spatial derivative *U*′(*x*), we find *u*_*j*_(*x, t*) = *U*_*j*_(*x*) + ε^1/2^ *U*′_*j*_(*x*) e^λ*t*^ is associated with a zero eigenvalue (λ = 0), corresponding to neutral stability. To see this, we plug it into the corresponding bump equation of Equation (1) in the absence of noise and interareal connections and examine the linearization

(9) $$\lambda U_{j^{\prime }}(x)=-U_{j^{\prime }}(x)+\int_{-\pi }^{\pi }w(x-y)f^{\prime }(U_{j}(y))U_{j^{\prime }}(y)\text{dy}.$$

Note, in the limit of infinite gain γ → ∞, a sigmoid *f* becomes the Heaviside (Equation 5), and

$$f^{\prime }(U(x))=\frac{\text{dH}(\text{U}(\text{x}))}{\text{dU}}=\frac{\delta (x-a)}{\left|U^{\prime }(a)\right|}+\frac{\delta (x+a)}{\left|U^{\prime }(a)\right|},$$

in the sense of the distributions. Equation (9) still hold in this case. Differentiating (Equation 7), and integrating by parts, we find

(10) $$\begin{array}{l} -U_{1}^{\prime }+w*[f^{\prime }(U_{1})U_{1}^{\prime }]=0,\\ -U_{2}^{\prime }+w*[f^{\prime }(U_{2})U_{2}^{\prime }]=0, \end{array}$$

where the boundary terms vanish due to periodicity of the domain [−π, π]. Thus, the right hand side of Equation (9) vanishes, and λ = 0 is the only eigenvalue corresponding to translating perturbations. Thus, either bump (in area 1 or 2) is neutrally stable to perturbations that shifts its position in either direction (rightwards or leftwards), since the bump in each area experiences no force from the other bump.

**Figure 1:**
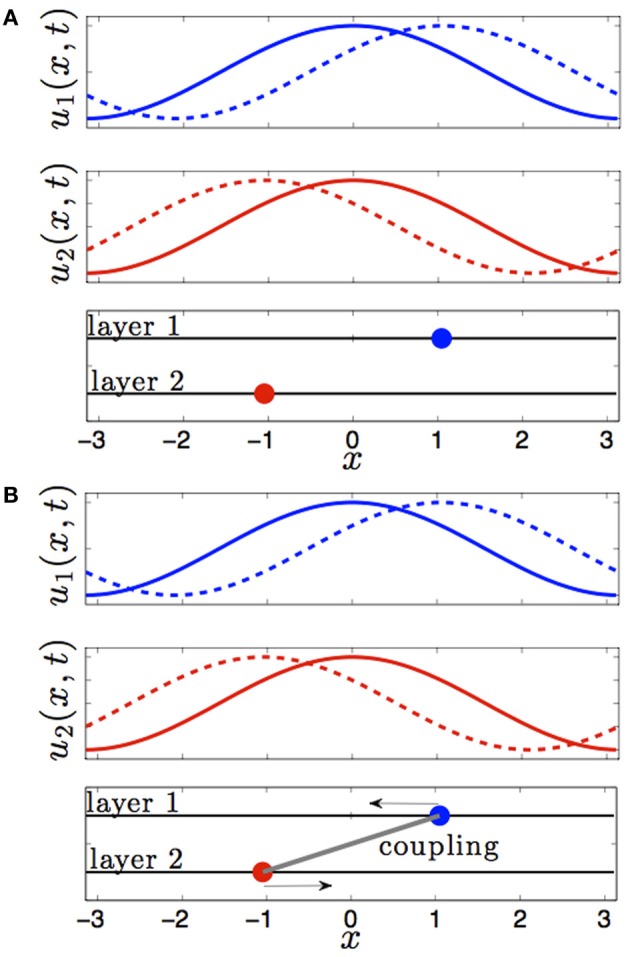
**Effect of interareal coupling on the stability of bumps to translating perturbations. (A)** In the absence of interareal coupling, bumps (solid) are neutrally stable to perturbations (dashed) that translate them in opposite directions. **(B)** In the presence of interareal coupling, bumps are linearly stable, as revealed by the negative eigenvalue in Equation (18), to perturbations that translate them in opposite directions.

This changes when we consider the effect of interareal connectivity. Once the two areas of Equation (1) are reciprocally coupled, bumps are stable to perturbations that translate them in opposite directions of one another (see Figure 1). Interareal connections act as a restoring force between the two positions of each bump. We will demonstrate this in the subsequent section by deriving a linear stochastic system for the position of either bump in the presence of small noise and weak interareal connectivity. The restorative nature of interareal connectivity is revealed by the negative eigenvalue associated with the interaction matrix (Equation 15) of our stochastic system, as shown in Equation (18).

### Noise-induced wandering of bumps

**Figure 2:**
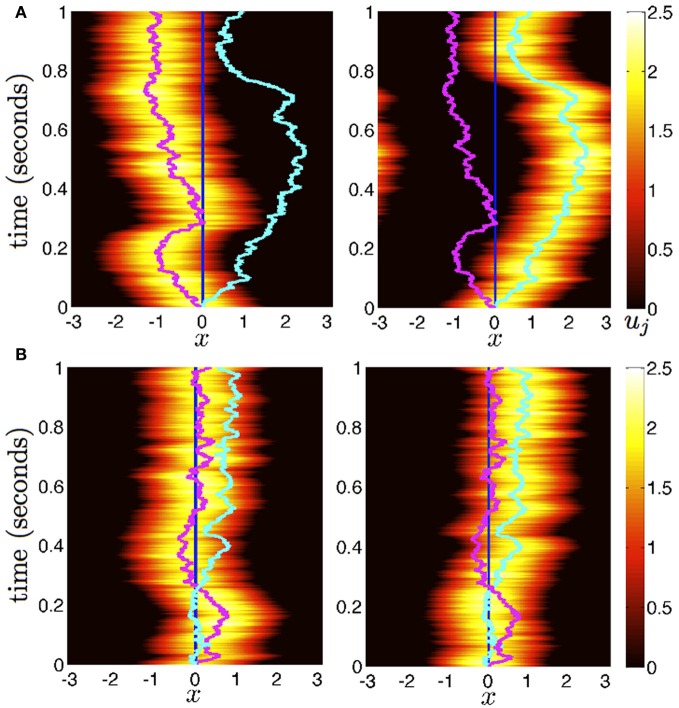
**Diffusion of bumps in the dual area stochastic neural field (Equation 1). (A)** Without interareal connections (*w*_12_ = *w*_21_ ≡ 0), each bump executes Brownian motion about the domain, due to stochastic forces. **(B)** In the presence of interareal connections $\sqrt{\varepsilon }w_{12}(x)=\sqrt{\varepsilon }w_{21}(x)=0.01(\cos(x)+1)$, the position of bump 1 (magenta) is attracted to the position of bump 2 (cyan) and vice versa. Due to the reversion of each bump to the position of the other, both bumps effectively wander the domain less. Local connectivity is described by the cosine (Equation 3); the firing rate function is Equation (5). Other parameters are threshold θ = 0.5 and noise amplitude ε = 0.025.

Now we consider the effects of small noise on the position of bumps in the presence of weak interareal connections. We start by presuming noise generates two distinct effects in the bumps (see Figure 2). First, noise causes both bumps to wander away from their initial positions, while still being pulled back into place by the bump in the other area. Bump position in areas 1 and 2 will be described by the time-varying stochastic variables Δ_1_(*t*) and Δ_2_(*t*). Second, noise causes fluctuations in the shape of both bumps, described by a correction Φ_*j*_. To account for this, we consider the ansatz

(11) $$\begin{array}{l} u_{1}=U_{1}(x-\Delta_{1}(t))+\varepsilon^{1/2}\Phi_{1}(x-\Delta_{1}(t),t)+\cdots \\ u_{2}=U_{2}(x-\Delta_{2}(t))+\varepsilon^{1/2}\Phi_{2}(x-\Delta_{2}(t),t)+\cdots \end{array}$$

Armero et al. (1998) originally developed this approach to analyze of front propagation in stochastic PDE models. In stochastic neural fields, it has been modified to analyze wave propagation (Bressloff and Webber, 2012a) and bump wandering (Kilpatrick and Ermentrout, 2013). Plugging the ansatz (Equation 11) into the system (Equation 1) and expanding in powers of ε^1/2^, we find that at 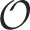
(1), we have the bump solution (Equation 7). Proceeding to 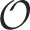
(ε^1/2^), we find

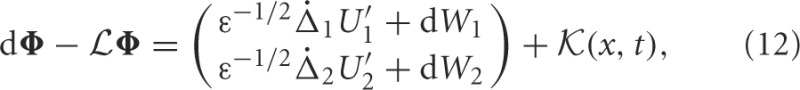



where 
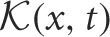
 is the 2 × 1 vector function

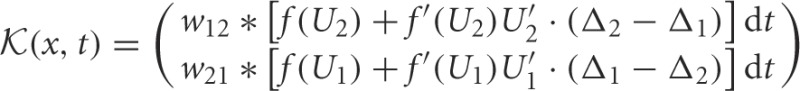



Φ = (Φ_1_(*x, t*), Φ_2_(*x, t*))^*T*^; and 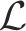 is the linear operator

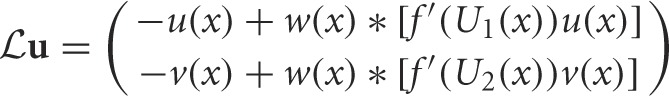



for any vector **u** = (*u*(*x*)*v*(*x*)^*T*^ of integrable functions. Note that the nullspace of 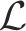 includes the vectors (*U*′_1_, 0)^*T*^ and (0, *U*′_2_)^*T*^, due to Equation (10). The last terms in the right hand side vector of Equation (12) arise due to interareal connections. We have linearized them under the assumption |Δ_1_ − Δ_2_| remains small, so

$$\begin{array}{l} f(U_{j}(x+\Delta_{k}-\Delta_{j}))\approx f(U_{j}(x))\\ \;+f^{\prime }(U_{j}(x))U_{j^{\prime }}(x)\cdot (\Delta_{k}-\Delta_{j}), \end{array}$$

where *j* = 1, 2 and *k* ≠ *j*. To make sure that a solution to Equation (12) exists, we require the right hand side is orthogonal to all elements of the null space of the adjoint 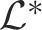, which is defined 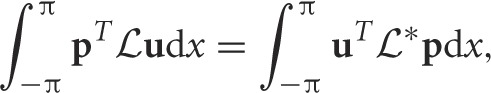 for any integrable vector **p** = (*p*(*x*)*q*(*x*))^*T*^. It then follows 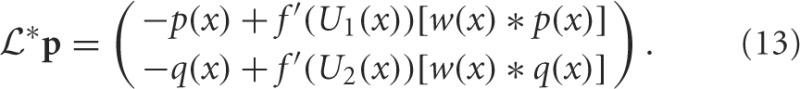


We can show that the nullspace of 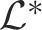 contains the vector **f**_1_ = (*f*′(*U*_1_)*U*′_1_, 0)^*T*^ by plugging it into Equation (13) to yield

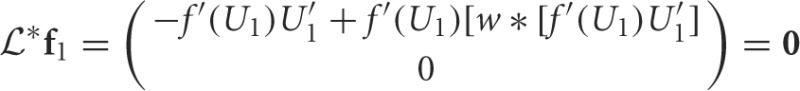



where **0** = (0, 0)^*T*^ and we use Equation (10). We can also show the nullspace of 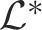 contains **f**_2_ = (0, *f*′(*U*_2_)*U*′_2_)^*T*^ in the same way. Thus, we can ensure Equation (12) has a solution by taking the inner product of both sides of Equation (12) with the two null vectors to yield

$$\begin{array}{l} \;\langle f^{\prime }(U_{1}){U^{\prime }}_{1},\varepsilon^{-1/2}{\dot{\Delta }}_{1}{U^{\prime }}_{1}+\text{d}W_{1}\\ +\;w_{12}*\left[f(U_{2})+f^{\prime }(U_{2}){U^{\prime }}_{2}\cdot (\Delta_{2}-\Delta_{1})\right]\text{d}t\rangle =0\\ \;\langle f^{\prime }(U_{2}){U^{\prime }}_{2},\varepsilon^{-1/2}{\dot{\Delta }}_{2}{U^{\prime }}_{2}+\text{d}W_{2}\\ +\;w_{21}*\left[f(U_{1})+f^{\prime }(U_{1}){U^{\prime }}_{1}\cdot (\Delta_{1}-\Delta_{2})\right]\text{d}t\rangle =0, \end{array}$$

where we define the inner product 〈*u*, *v*〉 = ∫^π^_−π_ *u*(*x*)*v*(*x*)d*x*. Therefore, the stochastic vector Δ(*t*) = (Δ_1_(*t*), Δ_2_(*t*))^*T*^ obeys the multivariate Ornstein–Uhlenbeck process

(14) dΔ(t)=KΔ(t)dt+dW(t)

where effects of interareal connections are described by the matrix

(15) $$K=\left(\begin{array}{cc} -\kappa_{1} & \kappa_{1}\\ \kappa_{2} & -\kappa_{2} \end{array}\right),$$

with

(16) $$\begin{array}{l} \kappa_{1}=\frac{\langle f^{\prime }(U_{1}){U^{\prime }}_{1},\varepsilon^{1/2}w_{12}*\left[f^{\prime }(U_{2}){U^{\prime }}_{2}\right]\rangle }{\langle f^{\prime }(U_{1}){U^{\prime }}_{1},{U^{\prime }}_{1}\rangle },\\ \kappa_{2}=\frac{\langle f^{\prime }(U_{2}){U^{\prime }}_{2},\varepsilon^{1/2}w_{21}*\left[f^{\prime }(U_{1}){U^{\prime }}_{1}\right]\rangle }{\langle f^{\prime }(U_{2}){U^{\prime }}_{2},{U^{\prime }}_{2}\rangle }, \end{array}$$

and (*w*_12_^*^ *f*(*U*_2_)) · *U*′_1_ and (*w*_21_^*^ *f*(*U*_1_)) · *U*′_2_ vanish upon integration since they are odd. Noise is described by the vector 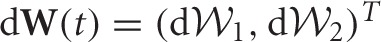 with

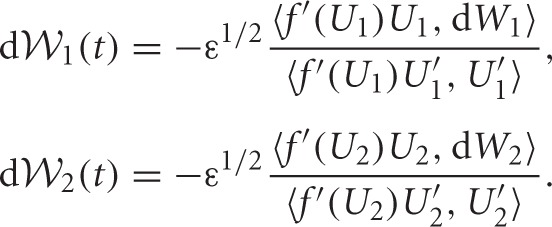



The white noise term **W** has zero mean 〈**W**(*t*)〉 = **0** and variance described by pure diffusion so 〈**W**(*t*)**W**^*T*^(*t*)〉 = **D***t* with

(17) $$D=\left(\begin{array}{cc} D_{1} & D_{c}\\ D_{c} & D_{2} \end{array}\right)$$

where the associated diffusion coefficients of the variance are

$$\begin{array}{l} D_{1}=\varepsilon \frac{\int_{-\pi }^{\pi }\int_{-\pi }^{\pi }F_{1}(x)F_{1}(y)C_{1}(x-y)\text{dxdy}}{\left[\int_{-\pi }^{\pi }F_{1}(x){U^{\prime }}_{1}(x)\text{dx}\right]},\\ D_{2}=\varepsilon \frac{\int_{-\pi }^{\pi }\int_{-\pi }^{\pi }F_{2}(x)F_{2}(y)C_{2}(x-y)\text{dxdy}}{\left[\int_{-\pi }^{\pi }F_{2}(x){U^{\prime }}_{2}(x)\text{dx}\right]}. \end{array}$$

where *F*_*j*_(*x*) = *f*′(*U*_*j*_(*x*))*U*′_*j*_(*x*) and covariance is described by the coefficient

$$D_{c}=\varepsilon \frac{\int_{-\pi }^{\pi }\int_{-\pi }^{\pi }F_{1}(x)f^{\prime }(U_{2}(y))F_{2}(y)C_{c}(x-y)\text{dxdy}}{\left[\int_{-\pi }^{\pi }F_{1}(x){U^{\prime }}_{1}(x)\text{dx}\right]\left[\int_{-\pi }^{\pi }F_{2}(x){U^{\prime }}_{2}(x)\text{dx}\right]}.$$

In the next section, we analyze this stochastic system (Equation 14), showing how coupling between areas can reduce the variability of the bump positions Δ_1_(*t*) and Δ_2_(*t*).

### Effect of coupling on bump position variance

To analyze the Ornstein–Uhlenbeck process (Equation 14), we start by diagonalizing the matrix **K** = **V Λ V**^−1^ using the eigenvalue decomposition

(18) $$\begin{array}{l} \;\Lambda =\left(\begin{array}{cc} 0 & 0\\ 0 & -\kappa_{1}-\kappa_{2} \end{array}\right),\\ \;V=\frac{1}{\kappa_{1}+\kappa_{2}}\left(\begin{array}{cc} 1 & \kappa_{1}\\ 1 & -\kappa_{2} \end{array}\right),\\ V^{-1}=\left(\begin{array}{cc} \kappa_{2} & \kappa_{1}\\ 1 & -1 \end{array}\right), \end{array}$$

such that Λ is the diagonal matrix of eigenvalues; columns of **V** are right eigenvectors; and rows of **V**^−1^ are left eigenvectors. Eigenvalues λ_1_, λ_2_ and eigenvectors **v**_1_, **v**_2_ inform us of the effect of interareal coupling on linear stability. The eigenvalue λ_1_ = 0 corresponds to the neutral stability of the positions (Δ_1_, Δ_2_)^*T*^ to translations in the same direction **v**_1_ = (1, 1)^*T*^. The negative eigenvalue λ_2_ = − (κ_1_ + κ_2_) corresponds to the linear stability introduced by interareal connections. The positions (Δ_1_, Δ_2_)^*T*^ revert to one another when perturbations translate them in opposite directions **v**_2_ = (κ_1_, −κ_2_)^*T*^.

Diagonalizing **K** = **V Λ V**^−1^ using Equation (18), we can compute the mean and variance of the vector Δ(*t*) given by Equation (14). First, note that the mean 〈Δ(*t*)〉 = e^**K***t*^ Δ(0) (Gardiner, 2003), which we can compute

$$\langle \Delta \rangle =\left(\begin{array}{c} \left(\kappa_{2}+\kappa_{1}{\text{e}}^{\lambda_{\text{2}}\text{t}})\Delta_{1}(0)+(\kappa_{1}-\kappa_{1}{\text{e}}^{\lambda_{\text{2}}\text{t}})\Delta_{2}(0\right)\\ \left(\kappa_{2}-\kappa_{2}{\text{e}}^{\lambda_{\text{2}}\text{t}})\Delta_{1}(0)+(\kappa_{1}+\kappa_{2}{\text{e}}^{\lambda_{\text{2}}\text{t}})\Delta_{2}(0\right) \end{array}\right)$$

using the diagonalization e^**K***t*^ = **V**e^Λ*t*^**V**^−1^. Since λ_2_ = −(κ_1_ + κ_2_) < 0,

$$\underset{t\;\to \;\infty }{\lim}\langle \Delta (t)\rangle =\left[\kappa_{2}\Delta_{1}(0)+\kappa_{1}\Delta_{2}(0)\right]\left(\begin{array}{c} 1\\ 1 \end{array}\right).$$

Thus, the means of Δ_1_(*t*) and Δ_2_(*t*) always relax to the same position in long time, due to the linear stability introduced by connections between areas. Under the assumption they both begin at Δ_1_(0) = Δ_2_(0) = 0, the covariance matrix is given (Gardiner, 2003)

(19) $$\langle \Delta (t)\Delta^{T}(t)\rangle =\int_{0}^{t}e^{\text{K}(t-s)}De^{{\text{K}}^{T}(t-s)}\text{d}s,$$

where **D** is the covariance coefficient matrix of the white noise vector **W**(*t*) given by Equation (17). To compute Equation (19), we additionally need the diagonalization **K**^*T*^ = (**V**^−1^)^*T*^ Λ **V**^*T*^, so e^**K**^*T*^ t^ = (**V**^−1^)^*T*^ e^Λ t^ **V**^*T*^. After multiplying and integrating (Equation 19), we find the elements of the covariance matrix

$$\langle \Delta (t)\Delta^{T}(t)\rangle =\left(\begin{array}{cc} \langle \Delta_{1}{\left(t\right)}^{2}\rangle & \langle \Delta_{1}(t)\Delta_{2}(t)\rangle \\ \langle \Delta_{1}(t)\Delta_{2}(t)\rangle & \langle \Delta_{2}{\left(t\right)}^{2}\rangle \end{array}\right)$$

are

(20) $$\langle \Delta_{1}{\left(t\right)}^{2}\rangle =D_{+}t+2\kappa_{1}r_{1}(t)+\frac{\kappa_{1}}{\kappa_{2}}r_{2}(t)$$

(21) $$\langle \Delta_{2}{\left(t\right)}^{2}\rangle =D_{+}t-2\kappa_{2}r_{1}(t)+\frac{\kappa_{2}}{\kappa_{1}}r_{2}(t)\;$$

$$\langle \Delta_{1}(t)\Delta_{2}(t)\rangle =D_{+}t+(\kappa_{1}-\kappa_{2})r_{1}(t)-r_{2}(t)$$

where the effective diffusion coefficients are

(22) $$D_{+}=\frac{\kappa_{2}^{2}D_{1}+2\kappa_{1}\kappa_{2}D_{c}+\kappa_{1}^{2}D_{2}}{{\left(\kappa_{1}+\kappa_{2}\right)}^{2}},$$

(23) $$D_{r}=\frac{\kappa_{2}D_{1}-\kappa_{1}D_{2}+(\kappa_{1}-\kappa_{2})D_{c}}{{\left(\kappa_{1}+\kappa_{2}\right)}^{2}},$$

(24) $$D_{-}=\frac{D_{1}-2D_{c}+D_{2}}{{\left(\kappa_{1}+\kappa_{2}\right)}^{2}},$$

so that *D*_+_ and *D*_−_ are variances of noises occurring along the eigendirections **v**_1_ and **v**_2_. The functions *r*_1_(*t*), *r*_2_(*t*) are exponentially saturating

$$\begin{array}{l} r_{1}(t)=\frac{D_{r}}{\kappa_{1}+\kappa_{2}}\left[1-{\text{e}}^{-(\kappa_{1}+\kappa_{2})t}\right],\\ r_{2}(t)=\frac{\kappa_{1}\kappa_{2}D_{-}}{2(\kappa_{1}+\kappa_{2})}\left[1-{\text{e}}^{-2(\kappa_{1}+\kappa_{2})t}\right]. \end{array}$$

The main quantities of interest to us are the variances (Equation 20) and (Equation 21) with which we can make a few observations concerning the effect of interareal connections on the variance of bump positions.

First, note the long term variance of either bump's position Δ_1_(*t*) and Δ_2_(*t*) will be the same, described by the averaged diffusion coefficient *D*_+_, since

(25) $$\underset{t\to \infty }{\lim}\langle \Delta_{1}{\left(t\right)}^{2}\rangle =\underset{t\to \infty }{\lim}\langle \Delta_{2}{\left(t\right)}^{2}\rangle =D_{+}t.$$

As the effective coupling strengths κ_*j*_ are increased, we can expect the variances 〈Δ_*j*_(*t*)^2^〉 approach these limits at faster rates since other portions of the variance decay at a rate proportional to |λ_2_| = κ_1_ + κ_2_.

Next, we study the case, across all times *t*, where connections between areas are the same (*w*_12_ ≡ *w*_21_ = *w*_*r*_) and noise within areas is identical (*D*_1_≡ *D*_2_ = *D*_*l*_), the mean reversion rates will be the same (κ_1_ = κ_2_ = κ) and terms in Equation (23) cancel so *D*_*r*_ = 0. Thus, the variances will be identical (〈Δ_1_(*t*)^2^〉 = 〈Δ_2_ (*t*)^2^〉 = 〈Δ(*t*)^2^〉) and

$$\langle \Delta {\left(t\right)}^{2}\rangle =\frac{D_{l}+D_{c}}{2}t+\frac{D_{l}-D_{c}}{8\kappa }\left[1-{\text{e}}^{\text{-4}\kappa \text{t}}\right].$$

This demonstrates the way in which correlated noise (*D*_*c*_) contributes to the variance. When noise within each area is shared (*D*_*c*_ → *D*_*l*_), there is no benefit to interareal coupling and 〈Δ(*t*)^2^〉 = *D*_*l*_*t* (see Kilpatrick and Ermentrout, 2013). However, when any noise is not shared between areas (*D*_*c*_ < *D*_*l*_), variance can be reduced by increasing coupling strength κ between areas. The variance 〈Δ(*t*)^2^〉 is monotone decreasing in κ since

$$\frac{\partial }{\partial \kappa }\langle \Delta {\left(t\right)}^{2}\rangle =\frac{D_{l}-D_{c}}{8}\frac{(1+4\kappa t){\text{e}}^{-\text{4}\kappa \text{t}}-\text{1}}{\kappa^{2}}\leq 0.$$

Inequality holds because (1 + 4 κ*t*) ≤ e^4 κ*t*^ is ensured by the Taylor series expansion of e^4 κ*t*^ when κ*t* > 0.

Thus, variance is minimized in the limit

(26) $$\underset{\kappa \to \infty }{\lim}\langle \Delta {\left(t\right)}^{2}\rangle =\frac{D_{l}+D_{c}}{2}t.$$

Therefore, strengthening interareal connections in *both* directions reduces the variance in bump position. On the other hand, in the limit of no interareal connections, we find lim_κ→0_〈Δ(*t*)^2^〉 = *D*_*l*_*t*, and the variance in a bump's position is determined entirely by local sources of noise.

Returning to asymmetric connectivity (κ_1_ ≠ κ_2_), we consider the case of feedforward connectivity from area 1 to 2 (*w*_12_≡ 0), κ_1_ = 0, so *D*_+_ = *D*_1_ and the formulas for the variances reduce to

$$\begin{array}{l} \langle \Delta_{1}{\left(t\right)}^{2}\rangle =\;D_{1}t,\\ \langle \Delta_{2}{\left(t\right)}^{2}\rangle =\;D_{1}t+\frac{2(D_{1}-D_{c})}{\kappa_{2}}\left[1-{\text{e}}^{-\kappa_{\text{2}}\text{t}}\right]\\ \;+\frac{D_{1}-2D_{c}+D_{2}}{2\kappa_{2}}\left[1-{\text{e}}^{-\text{2}\kappa_{\text{2}}\text{t}}\right], \end{array}$$

so the pure diffusive term of both variances is wholly determined by the local noise of area 1. Then, only the position of the bump in area 2 possesses additional mean-reverting fluctuations in its position, which arise from local sources of noise that force it away from the position of the bump in area 1. In this situation, the variance of the bump in area 2's position is minimized when

$$\underset{\kappa_{2}\to \infty }{\lim}\langle \Delta_{1}{\left(t\right)}^{2}\rangle =\underset{\kappa_{2}\to \infty }{\lim}\langle \Delta_{2}{\left(t\right)}^{2}\rangle =D_{1}t.$$

Comparing this with Equation (26) we see that, since *D*_*c*_ ≤ *D*_1_, the variances 〈Δ_*j*_(*t*)^2^〉 will always be higher in this case than in the case of very strong reciprocal coupling between both areas. Averaging information and noise between both areas decreases positional variance as opposed to one area simply receiving noise and information from another. Similar results have been recently identified in the context of studying synchrony of reciprocally coupled noisy oscillators (Ly and Ermentrout, 2010).

One important caveat is that if area 1 has more noise than area 2, the weighting of reciprocal connectivity, κ_1_ and κ_2_, should be balanced to minimize the variance. If the average diffusion coefficient *D*_+_ is weighted too heavily with the area having the larger variance, the area with less intrinsic noise can end up noisier than it would be without reciprocal connectivity. To see this in the extreme case feedforward coupling, note that if *D*_2_ < *D*_1_, then *D*_2_*t* < *D*_1_*t* < 〈Δ_2_(*t*)^2^〉. Thus, the variance of Δ_2_(*t*) increases as opposed to the uncoupled case where 〈Δ_2_(*t*)^2^〉 = *D*_2_*t*.

We now derive the optimal weighting of κ_1_ and κ_2_ to minimize the long term variance (Equation 25) for general asymmetric connectivity, in the absence of correlated noise *D*_*c*_ = 0. To do so, we fix κ_2_ and find the κ_1_ that minimizes *D*_+_, which happens to be

$$\kappa_{1}=\kappa_{2}\frac{D_{1}}{D_{2}}.$$

Thus, for identical noise *D*_1_ = *D*_2_, setting κ_1_ = κ_2_ minimizes *D*_+_. For much stronger noise in area 2 (*D*_2_ » *D*_1_), κ_1_ should be made relatively small. In the case of noise correlations between areas (*D*_*c*_ > 0), the optimal value of κ_1_ that minimizes (Equation 25) is

$$\kappa_{1}=\kappa_{2}\frac{D_{1}-D_{c}}{D_{2}-D_{c}}.$$

### Calculating the stochastic motion of bumps

We now compute the effective variances (Equation 20) and (Equation 21), considering the specific case of Heaviside firing rate functions (Equation 5), cosine synaptic weights (Equation 3) and (Equation 4). Doing so, we can compare our asymptotic results to those computed from numerical simulations. We compute the mean reversion terms κ_1_ and κ_2_ by noting the spatial derivative of each bump will be *U*′_1_(*x*) = *U*′_2_(*x*) = −2sin*a* sin*x* and the null vector components are

$$f^{\prime }(U_{j}(x)){U^{\prime }}_{j}(x)=\delta (x+a)-\delta (x-a).$$

for *j* = 1, 2. Plugging these formulae into Equation (16), we find κ_1_ = ε^1/2^*M*_1_ and κ_2_ = ε^1/2^*M*_2_.

We first consider the case of uncorrelated noise between areas, so *c*_*c*_ ≡ 0, meaning *D*_*c*_ = 0. We can compute the diffusion coefficients associated with the local noise in each area assuming cosine spatial correlations

(27) $$D_{1}=\frac{c_{1}\varepsilon }{2+2\sqrt{1-\theta^{2}}},\;D_{2}=\frac{c_{2}\varepsilon }{2+2\sqrt{1-\theta^{2}}}.$$

We can then compute Equations (20) and (21) directly, for the case of no noise correlations between areas, by plugging in Equation (27).

For symmetric connections between areas, κ = ε^1/2^ *M*_1_ = ε^1/2^*M*_2_, as well as identical noise, *c*_1_ = *c*_2_ = 1, we have 〈Δ_1_(*t*)^2^〉 = 〈Δ_2_(*t*)^2^〉 = 〈Δ(*t*)^2^〉 and

(28) $$\langle \Delta {\left(t\right)}^{2}\rangle \text{​ }=\text{ ​}\frac{\varepsilon t}{4(1+\sqrt{1-\theta^{2}})}\text{ ​}+\text{ ​}\frac{\varepsilon }{16(1+\sqrt{1-\theta^{2}})\kappa }\left[1-{\text{e}}^{-\text{4}\kappa \text{t}}\right]\text{​}.$$

**Figure 3:**
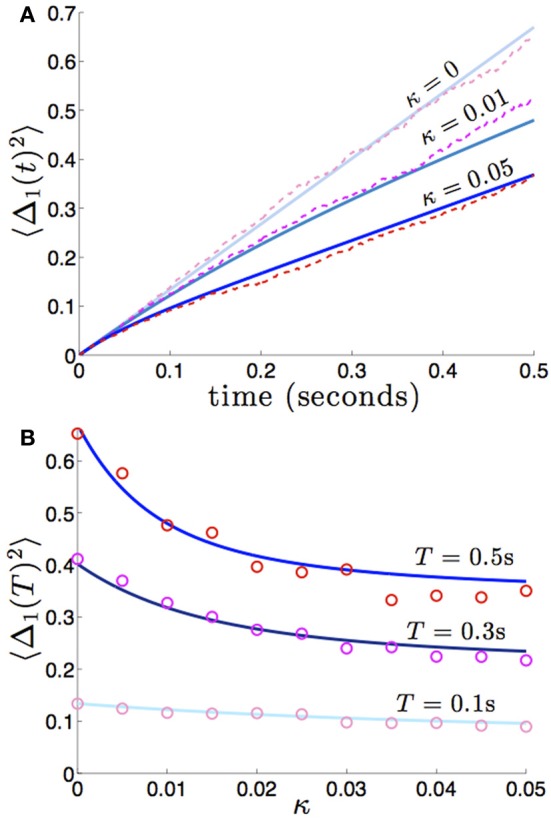
**Variance in the position of bumps as computed numerically (red shades) and from theory (blue shades) using Equation (28)**. Coupling between areas is symmetric $\sqrt{\varepsilon }w_{12}(x)=\sqrt{\varepsilon }w_{21}(x)=\kappa (\cos(x)+1)$, so 〈Δ_1_(*t*)^2^〉 = 〈Δ_2_(*t*)^2^〉, and there is no shared noise (*c*_*c*_ = 0). **(A)** The increase in variance is slower for stronger amplitudes of interareal coupling κ. Notice variance climbs sublinearly for κ > 0, due to the mean-reversion caused by coupling. **(B)** Variance drops considerably more over low values of κ that over high values. Other constituent functions and parameters are the same as in Figure 2.

**Figure 4:**
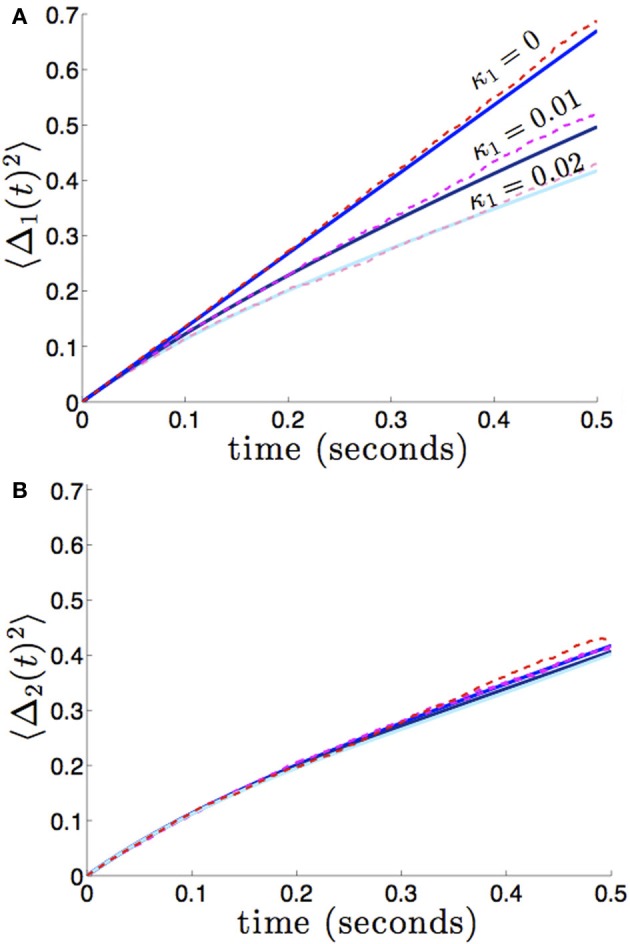
**Variance in the position of bumps as it depends on asymmetric reciprocal connectivity (κ_1_ ≠ κ_2_) when noise in each area is independent and identical (*c*_1_ = *c*_2_ = 1)**. Fixing κ_2_ = 0.02 and varying κ_1_, we find **(A)** the variance 〈Δ_1_(*t*)^2^ of bump 1 decreases as coupling from area 2 to 1 (κ_1_) increases; **(B)** variance 〈Δ_2_(*t*)^2^〉 of bump 2 remain relatively unchanged. Other constituent functions and parameters are the same as in Figure 2.

**Figure 5:**
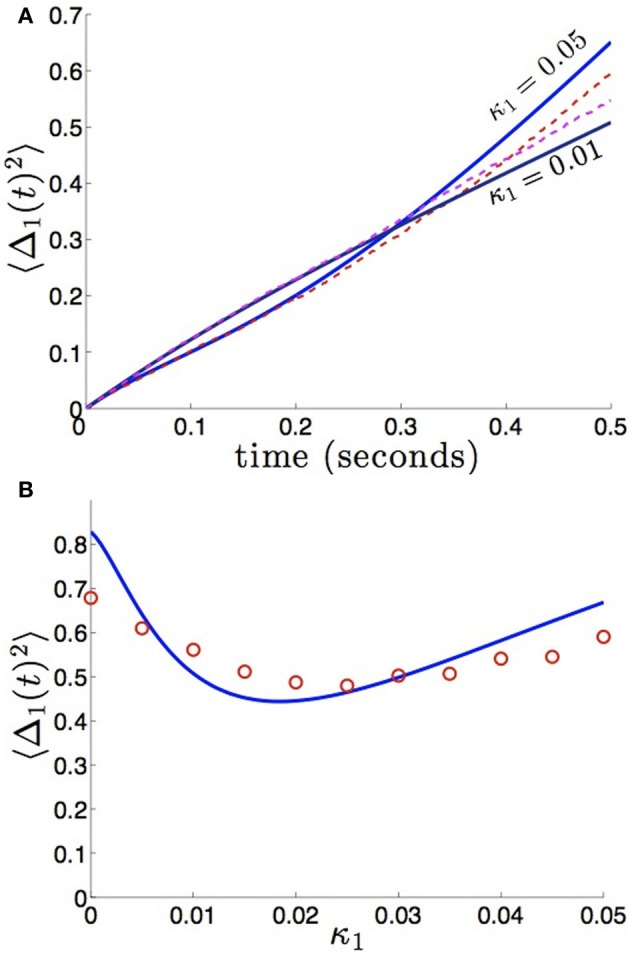
**Bump position variance depends non-monotonically on asymmetric connectivity strength. (A)** For κ_2_ = 0.01 and high enough values of coupling (κ_1_ = 0.05), variance 〈Δ_1_(*t*)^2^〉 scales more quickly than for symmetric coupling (κ_1_ = 0.01). Layer 1 is being sourced by the noisier area 2. **(B)** Non-monotonic dependence of variance 〈Δ_1_(*t*)^2^〉 on projection strength from area 2 to area 1 κ_1_ is shown for fixed time *T* = 50 and κ_2_ = 0.01 fixed. Amplitude of noise in area 2 is twice that of area 1 (*c*_1_ = 1 and *c*_2_ = 2). Other constituent functions and parameters are the same as in Figure 2.

We compare the formula (28) to results we obtain from numerical simulations in Figure 3, finding our asymptotic formula (28) matches quite well. In addition, we compare our results for general (possibly asymmetric) reciprocal connectivity to results from numerical simulations in Figure 4. We also show in Figure 5, as predicted, when κ_2_ is held fixed, there is a finite optimal value of κ_1_ that minimizes variance 〈Δ_1_ (*t*)^2^〉. Therefore, reciprocal connectivity in multi-area networks should be balanced, in order to minimize positional variance of the stored bump.

Next, we consider the case of correlated noise between areas, so *c*_*c*_ > 0, meaning *D*_*c*_ > 0. In this case, the covariance terms in *D*_+_ and *D*_−_ are non-zero. We can thus compute the diffusion coefficient associated with correlated noise

$$D_{c}=\frac{c_{c}\varepsilon }{2+2\sqrt{1-\theta^{2}}}.$$

**Figure 6:**
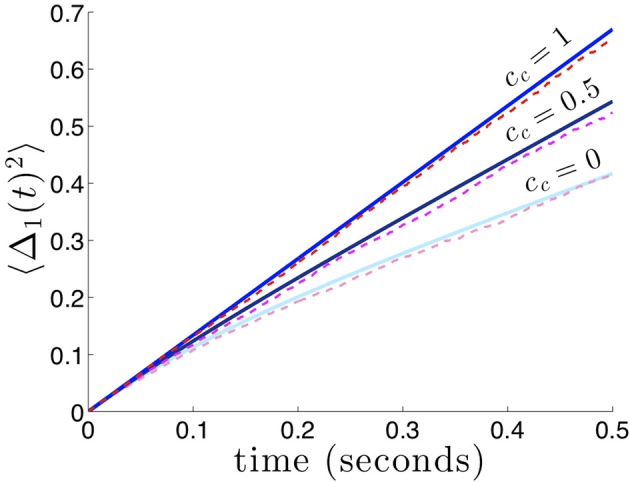
**Variance in the position of bumps as noise correlation between areas is increased**. Numerically computed variance (red shades) match theoretical curves from Equation (29), blue shades, very well. Reciprocal connectivity reduces variability the most when there is no correlated noise (*c*_*c*_ = 0) between areas. As the shared noise between areas increased is amplitude (*c*_*c*_ = 0.5, 1), the advantage of reciprocal connectivity is diminished. When *c*_*c*_ = 1 changing κ does not affect the variance 〈Δ(*t*)^2^〉 (see formula (29) in the limit *c*_*c*_ → 1). Other constituent functions and parameters are the same as in Figure 2.

In the case of symmetric connections between areas, κ = ε^1/2^*M*_1_ = ε^1/2^*M*_2_, and identical internal noise, *c*_1_ = *c*_2_ = 1, we have 〈Δ_1_(*t*)^2^〉 = 〈Δ_2_(*t*)^2^〉 = 〈Δ(*t*)^2^〉 and

(29) $$\langle \Delta {\left(t\right)}^{2}\rangle =\frac{(1+c_{c})\varepsilon }{4\left(1+\sqrt{1-\theta^{2}}\right)}t+\frac{(1-c_{c})\varepsilon }{16\left(1+\sqrt{1-\theta^{2}}\right)\kappa }\left[1-{\text{e}}^{-\text{4}\kappa \text{t}}\right],$$

which reflects the fact that interareal connections do not reduce variability as much when there are strong noise correlations *c*_*c*_ between areas. We demonstrate the accuracy of the theoretical calculation (Equation 29) as compared to numerical simulations in Figure 6. Numerical simulations also reveal the fact that stronger noise correlations between areas diminish the effectiveness of interareal connections at reducing bump position variance.

### Reduction of bump wandering in multiple areas

We now examine the effect of interareal connections in networks with more than two areas using the system (Equation 6). As with the dual area network without noise or interareal connectivity, stationary bump solutions take the form (*u*_1_, …, *u*_*N*_) = (*U*_1_(*x*),…, *U*_*N*_(*x*)), and translation invariance let us to set all bump peaks to be located at *x* = 0 so

(30) $$U_{j}=w*f(U_{j}),\;j=1,\ldots ,N.$$

As before, we presume *w*_*jj*_ = *w*, and relaxing this assumption does not dramatically alter our results. Linear stability analysis of bumps proceeds along similar lines to the dual area network, so we omit those calculations and summarize the results. In the absence of interareal connections, each bump is neutrally stable to perturbation in either direction. In the presence of interareal connections, all bumps are only neutrally stable to translations that move them all in the same direction. Therefore, networks with more areas provide more perturbation cancelations.

**Figure 7:**
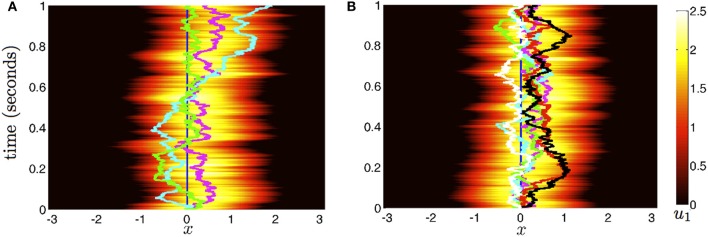
**Stochastic evolution of bump position in multi-area networks. (A)** With weak coupling $\sqrt{\varepsilon }w_{jk}(x)=0.01(\cos(x)+1)$ for *j* ≠ *k*) between *N* = 3 areas, the position of bumps 1 (magenta), 2 (cyan), and 3 (green) reverts to one another. We show only the evolution of activity *u*(*x, t*) in area 1. **(B)** For *N* = 6 areas and the same interareal coupling, the reduction in bump wandering is even more considerable. The trajectories of bumps in all areas (colored lines) stay close together. All other parameters are as in Figure 2.

To study how noise and interareal connections affect the trajectory of bump positions, we again note noise causes all bumps to wander away from their initial position, while being pulled back into place by projections from other areas (see Figure 7). The position of the bump in area *j* is described by the stochastic variable Δ_*j*_. Noise also causes fluctuations in the shape of both bumps, which is described by the correction term Φ_*j*_. Therefore, we presume the resulting state of the system satisfies the ansatz

$$u_{j}=U_{j}(x-\Delta_{j}(t))+\varepsilon^{1/2}\Phi_{j}(x-\Delta_{j}(t),t)+\cdots ,$$

where *j* = 1,…, *N*. Plugging this ansatz into Equation (6) and expanding in powers of ε^1/2^, we find that at 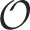
(1), we simply have the system of Equation (30) for the bump solutions. Proceeding to 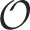
(ε^1/2^), we find 
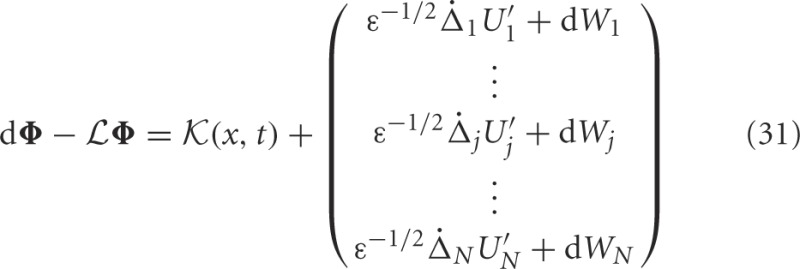
 where 
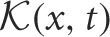
 is an *N* × 1 vector whose *j*th entry is 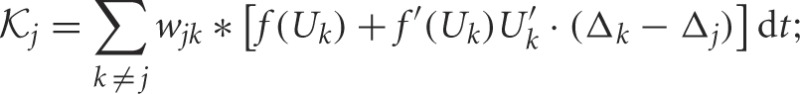 Φ = (Φ_1_(*x, t*), …, Φ_*N*_(*x, t*))^*T*^; and 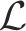 is the linear operator 
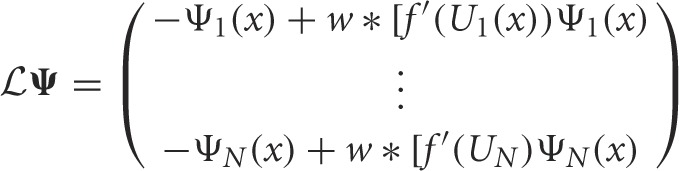
 for any integrable vector Ψ = (Ψ_1_(*x*),…, Ψ_*N*_(*x*))^*T*^. The nullspace of 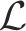 is spanned by the vectors (*U*′_1_, 0, …, 0)^*T*^; (0, *U*′_2_, 0, …, 0)^*T*^; …; and (0, …, 0, *U*′_*N*_)^*T*^, which can be seen by differentiating (Equation 30). The last terms on the right hand side of Equation (31) arise due to interareal connections. We have linearized them under the assumption that |Δ_*k*_ − Δ_*j*_| remains small for all *j*, *k*. To ensure a solution to Equation (31), we require the right hand side is orthogonal to all elements of the null space of the adjoint operator 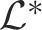. The adjoint is defined with respect to the inner product 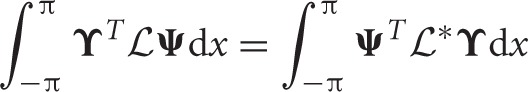 where ϒ = (ϒ_1_(*x*),…,ϒ_*N*_(*x*))^*T*^ is integrable. It then follows 
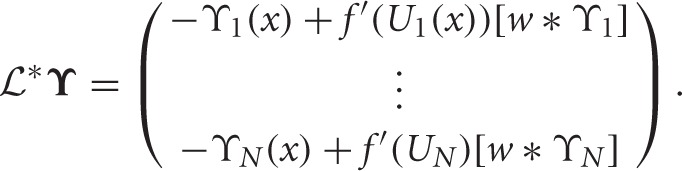


The nullspace of 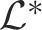 contains the vectors (*f*′(*U*_1_)*U*′_1_, 0, …, 0)^*T*^; (0, *f*′(*U*_2_)*U*′_2_, 0, … 0)^*T*^; …; and (0, …, 0, *f*′(*U*_*N*_)*U*_*N*_′), which can be shown by applying 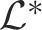 to them and using the formula generated by differentiating (Equation 30). Thus, to be sure (Equation 31) has a solution, we take the inner product of both sides of the equation with all *N* null vectors and isolate dΔ_*j*_ terms to yield the multivariate Ornstein–Uhlenbeck process

(32) dΔ(t)=KΔ(t)dt+dW(t),

where effects of interareal connections are described by the matrix **K** ∈ ℝ^*N*×*N*^ where the diagonal and off-diagonal entries are given

$$K_{jj}=-\sum_{k\neq j}\kappa_{jk},\;K_{jk}=\kappa_{jk}$$

for *j* = 1,…, *N* and *k* ≠ *j*, where

$$\kappa_{jk}=\frac{\langle f^{\prime }(U_{j}){U^{\prime }}_{j},\varepsilon^{1/2}w_{jk}*[f^{\prime }(U_{k}){U^{\prime }}_{k}]\rangle }{\langle f^{\prime }(U_{j}){U^{\prime }}_{j},{U^{\prime }}_{j}\rangle },$$

and we have used the fact that *w*_jk_^*^ *f*(*U*_*k*_) · *U*′_*j*_ is an odd function for all *j, k*, so they vanish on integration. Stochastic forces are described by the vector 
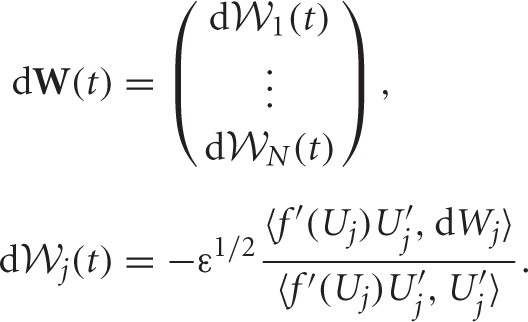


The white noise vector **W**(*t*) has zero mean 〈**W**(*t*)〉 = **0**, and covariance matrix 〈**W**(*t*)**W**^*T*^(*t*)〉 = **D***t* where associated coefficients of the matrix **D** are

$$D_{jj}=\varepsilon \frac{\int_{-\pi }^{\pi }\int_{-\pi }^{\pi }F_{j}(x)F_{j}(y)C_{j}(x-y)\text{dxdy}}{{\left[\int_{-\pi }^{\pi }F_{j}(x){U^{\prime }}_{j}(x)\text{dx}\right]}^{2}}.$$

where *F*_*j*_(*x*) = *f*′(*U*_*j*_(*x*))*U*′_*j*_(*x*), which describe the variance within an area and

$$D_{jk}=\varepsilon \frac{\int_{-\pi }^{\pi }\int_{-\pi }^{\pi }F_{j}(x)F_{k}(y)C_{jk}(x-y)\text{dxdy}}{\left[\int_{-\pi }^{\pi }F_{j}(x){U^{\prime }}_{j}(x)\text{dx}\right]\left[\int_{-\pi }^{\pi }F_{k}(x){U^{\prime }}_{k}(x)\text{dx}\right]},$$

which describes covariance between areas. Since correlations are symmetric *C*_*jk*_(*x*) = *C*_*kj*_(*x*) for all *j*, *k*, then *D*_*jk*_ = *D*_*kj*_ for all *j, k*.

A detailed analysis of the linear stochastic system (Equation 32) is difficult without some knowledge of the entries κ_*jk*_. However, we can make a few general statements. We note that all eigenvalues of **K** must have negative real part or be zero, due to the Gerschgorin circle theorem (Feingold and Varga, 1962), which states that all eigenvalues a matrix **K** must lie in one of the disks with center *K*_*jj*_ and radius ∑_*k≠j*_ |*K*_*jk*_|. Since *K*_*jj*_ = − ∑_*k≠j*_ κ_*jk*_ and *K*_*jk*_ = κ_*jk*_, then

(33) $$K_{jj}+\sum_{k\neq j}K_{jk}=-\sum_{k\neq j}\kappa_{jk}+\sum_{k\neq j}\left|\kappa_{jk}\right|=0$$

is the maximal possible eigenvalue, since κ_*jk*_ ≥ 0 for all *j, k*. Therefore, we expect *N* eigenpairs λ_*j*_, **v**_*k*_ associated with **K**, where λ_*N*_ ≤ λ_*N* − 1_ ≤ … ≤ λ_2_ ≤ λ_1_ = 0. This means we can perform the diagonalization **K** = **V** Λ **V**^−1^, where Λ is the diagonal matrix of eigenvalues; columns of **V** are right eigenvectors; and rows of **V**^−1^ are left eigenvectors. Therefore, we can decompose the stochastic solution to Equation (32), when **Δ**(0) = **0** as

$$\Delta (t)=\int_{0}^{t}e^{K(t-s)}\text{d}W(s)=\int_{0}^{t}V{\text{e}}^{\Lambda (\text{t-s})}V^{\text{-1}}\text{d}W(\text{s}),$$

Thus, as we expect, any stochastic fluctuations in Equation (32) will be integrated or decay over time due to the exponential filters e^λ_*j*_(*t − s*)^. In addition, when Δ(0) = **0** the covariance matrix can be computed as

(34) $$\langle \Delta \left(t\right)\Delta^{T}\left(t\right)\rangle =\int_{0}^{t}e^{\text{K}(t-s)}{\text{De}}^{{\text{K}}^{T}(t-s)}\text{d}s,$$

where **D** is the matrix of diffusion coefficients for the covariance 〈**W**(*t*) **W**^*T*^(*t*)〉. We now compute the covariance in the specific case of symmetric connectivity.

In the case of symmetric connectivity between areas, *w*_*jk*_ = *w*_*r*_ for all *j* ≠ *k*, so κ_*jk*_ = κ for all *j* ≠ *k*. Effects of connectivity between areas are described by the symmetric matrix

$$K=\kappa J_{N}-N\kappa I$$

where *J*_*N*_ is the *N* × *N* matrix of ones and *I* is the identity. The eigenvalues of *J*_*N*_ are *N*, with multiplicity one, and zero, with multiplicity *N* − 1. Thus, the largest eigenvalue of **K** = κ*J*_*N*_ − *N* κ*I* is λ_1_ = 0 with associated eigenvector **v**_1_ = (1,…, 1)^*T*^. All other eigenvalues are λ_*j*_ = −*N*κ for *j* ≥ 2, with associated eigenvectors **v**_*j*_ = **e**_1_ − **e**_*j*_, where *j* = 2, …, *N* and **e**_*j*_ is the unit vector with a one in the *j*th row and zeros elsewhere. Our diagonalization of the symmetric matrix **K** = **K**^*T*^ = **V** Λ **V**^−1^ then involves the diagonal matrix Λ of eigenvalues λ_*j*_; the symmetric matrix **V** whose columns **v**_*j*_ are right eigenvectors; and the symmetric matrix **V**^−1^ whose rows are left eigenvectors. The matrix **V**^−1^ takes the form

$$V^{-1}=\frac{1}{N}\left(\begin{array}{cccc} 1 & 1 & \cdots & \\ 1 & -(N-1) & 1 & \cdots \\ & & \ddots & 1\\ 1 & \cdots & 1 & -(N-1) \end{array}\right).$$

We can thus compute the covariance using the diagonalization e^**K***t*^ = e^**K**^*T*^*_t_*^ = **V** e^Λ*t*^**V**^−1^. In addition, we will assume each area receives noise with identical statistics (*D*_jj_ = *D*_*l*_) and there are identical noise correlations between areas (*D*_*jk*_ = *D*_*c*_ for *j* ≠ *k*), so **D** = (*D*_*l*_ − *D*_*c*_)*I* + *D*_*c*_*J*_*N*_. Multiplying and integrating (Equation 34), we find the diagonal entries (variances) of 〈Δ(*t*)Δ^*T*^(*t*)〉 are

(35) $$\langle \Delta_{j}{\left(t\right)}^{2}\rangle =\frac{D_{l}+(N-1)D_{c}}{N}t+\frac{\left(N-1)(D_{l}-D_{c}\right)}{2N^{2}\kappa }\left[1-{\text{e}}^{-\text{2N}\kappa \text{t}}\right],$$

and the off-diagonal entries (true covariances) are

$$\langle \Delta_{j}(t)\Delta_{k}(t)\rangle =\frac{D_{l}+(N-1)D_{c}}{N}t-\frac{\left(D_{l}-D_{c}\right)}{2N^{2}\kappa }\left[1-{\text{e}}^{-\text{2N}\kappa \text{t}}\right].$$

As revealed by the diffusive term in Equation (35), the system still possesses a rotational symmetry, given by the action of rotating all the bumps in the same direction. Thus, the component of noise in this direction is not damped out by coupling. Thus, note that the long term variance of any bump's position Δ_*j*_(*t*) will be approximately described by the averaged diffusion

$$\underset{t\to \infty }{\lim}\langle \Delta_{j}{\left(t\right)}^{2}\rangle =\frac{D_{l}+(N-1)D_{c}}{N}t.$$

As the strength of coupling κ or number of areas *N* is increased, the variances 〈Δ_*j*_(*t*)^2^〉 approach this limit at a faster rate, since the other portions of variance decay at a rate proportional to |λ_2_| = *N*κ. Note also that in the limit *D*_*c*_ → *D*_*l*_, effects of coupling are negligible and the long term variance of each bump is determined by the diffusion introduced by its area's internal noise.

Returning to study the full variance Equation (35) for symmetric coupling and noise, we make a few observations. First, in the limit of purely correlated noise across areas (*D*_*c*_ → *D*_*l*_), interareal connections have no effect, and 〈Δ_*j*_(*t*)^2^〉 = *D*_*l*_*t* for all areas and arbitrary coupling strength. However, if there is any independent noise in each area (*D*_*c*_ < *D*_*l*_), variance 〈Δ_*j*_(*t*)^2^〉 can always be reduced further by increasing coupling strength or the number of areas since

$$\frac{\text{d}}{\text{d}\kappa }\langle \Delta_{j}{\left(t\right)}^{2}\rangle =\frac{\left(N-1)(D_{l}-D_{c}\right)}{2N^{2}}\times \frac{(1+2N\kappa ){\text{e}}^{-\text{2N}\kappa \text{t}})-\text{1}}{\kappa^{2}}\leq 0,$$

where inequality (1 + 2*N*κ*t*) ≥ e^2*N*κ*t*^ holds due to the Taylor expansion of e^2*N*κ*t*^ when *N*κ*t* ≥ 0, and

$$\begin{array}{l} \frac{\text{d}}{\text{dN}}\langle \Delta_{j}{\left(t\right)}^{2}\rangle =-\frac{D_{l}-D_{c}}{N^{2}}\\ \;+\frac{D_{l}-D_{c}}{2N^{3}\kappa }\left[2(1+N\kappa t){\text{e}}^{-\text{2N}\kappa \text{t}}-\text{N}\right]\leq 0 \end{array}$$

when *N* ≥ 2, since *D*_*l*_ ≥ *D*_*c*_ and due to the Taylor expansion of e^2*N*κ*t*^. Note, we have temporarily treated *N* as a continuous variable. Thus, we know the variance 〈Δ_*j*_(*t*)^2^〉 to decrease with increasing κ and expect it to decrease with increasing *N*.

**Figure 8:**
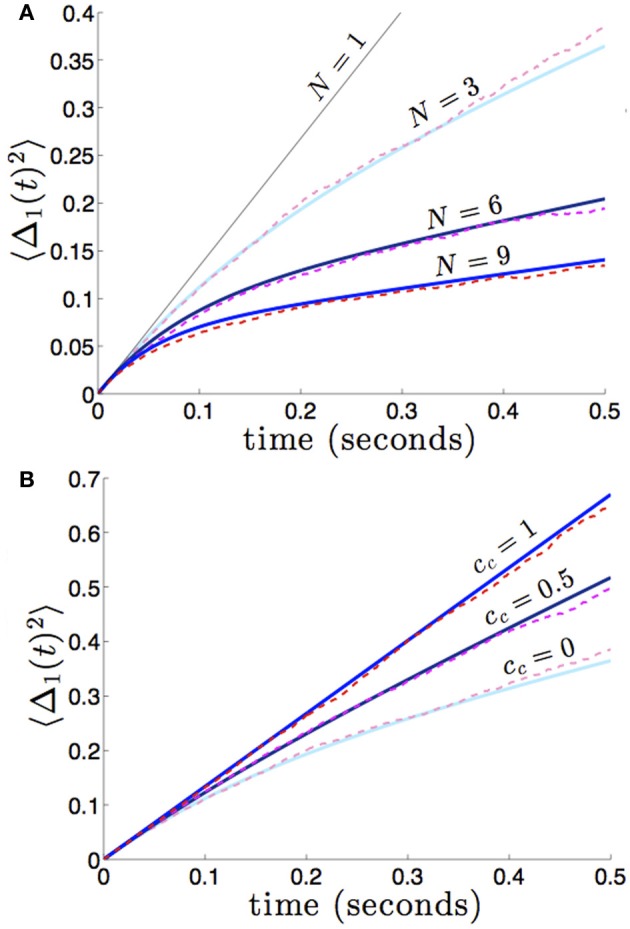
**(A)** Variance in the position of the bump in the first area 〈Δ_1_(*t*)^2^〉 builds up more slowly in networks with more areas *N*, and we expect similar behavior in all other areas. Fixing the strength of interareal connections, $\sqrt{\varepsilon }w_{jk}(x)=0.01(\cos(x)+1)$ for *j* ≠ *k*, we see that varying *N* decreases the variance 〈Δ_*j*_(*t*)^2^〉. **(B)** As in dual area networks, increasing the level of noise correlations between areas diminishes the effectiveness of interareal connectivity as a noise cancelation mechanism. Other parameters are as in Figure 2.

We can compute the variance 〈Δ_*j*_(*t*)^2^〉 explicitly in the case of Heaviside firing rate functions (Equation 5), cosine synaptic weights (Equation 3) and (Equation 4). With these assumptions, as well as there being identical noise to all areas (*c*_*jj*_ = 1 for all *j*, *c*_*jk*_ = *c*_*c*_ for *j* ≠ *k*), we find

$$D_{l}=\frac{\varepsilon }{2+2\sqrt{1-\theta^{2}}},\;D_{c}=\frac{c_{c}\varepsilon }{2+2\sqrt{1-\theta^{2}}},$$

so that

(36) $$\langle \Delta_{j}{\left(t\right)}^{2}\rangle =\frac{(1+(N-1)c_{c})\varepsilon }{2N(1+\sqrt{1-\theta^{2}})}t+\frac{(1-c_{c})\varepsilon }{4N^{2}\kappa }\left[1-{\text{e}}^{-\text{2N}\kappa \text{t}}\right],$$

which reflects the fact that increasing the number of areas will decrease variability, when noise between areas is not too strongly correlated. We demonstrate the accuracy of this formula (36) in Figure 8. In numerical simulations, as predicted by our asymptotic calculations, the variance scales more slowly in time in networks with more areas.

## Discussion

We have shown that interareal coupling in multi-area stochastic networks can reduce the diffusive wandering of bumps. Since bump attractors offer a well studied model of persistent activity underlying spatial working memory (Compte et al., 2000), our results provide a novel suggestion for how the memory networks may reduce error. Our calculations have exploited a small noise approximation for the position of the bump in each area (Armero et al., 1998; Bressloff and Webber, 2012a). Assuming connectivity between areas is weak, we have shown the equations describing bump positions reduce to a multivariate Ornstein–Uhlenbeck process. In this formulation, we find interareal connectivity stabilizes all but one eigendirection in the space of bump position movements. Neutral stability does still exist, so stochastic forces that move bumps in all areas in the same direction do not decay away. However, sources of noise that force bumps in opposite directions create bump movements that will decay with time. Thus, interareal connectivity provides a noise cancelation mechanism that operates by stabilizing the bumps in each area to stochastic forces that push them in opposite directions. (Polk et al., 2012) recently explored noise correlation statistics in persistent state networks that reduce wandering. Our work complements these results by studying synaptic architectures that limit persistent state diffusion.

Storing spatial working memories with neural activity that spans multiple brain areas does serve other purposes than potential noise cancelation. Delayed response tasks that lead to limb motion can generate persistent activity in the parietal cortex (Colby et al., 1996; Pesaran et al., 2002) so that motor responses can be readily executed. In addition, superior colliculus demonstrates sustained activity (Basso and Wurtz, 1997), which is an area also thought to underlie directed behavioral responses. Therefore, activity is distributed between areas providing short term information storage, like prefrontal cortex (Goldman-Rakic, 1995), and those responsible for motor responses and/or behavior. An additional effect of this delegation of activity is that reciprocal connections between areas may provide noise cancelation during the storage period of working memory. However, our work suggests distributing working memory-serving neural activity between areas that receive strongly correlated noise will not provide as effective cancelation.

Our work should be contrasted with several other results concerning the stabilization of networks that encode a continuous variable (Koulakov et al., 2002; Goldman et al., 2003; Cain and Shea-Brown, 2012; Kilpatrick et al., 2013). Pure integrators, which are usually line attractors, are notoriously fragile to parametric perturbations, so (Koulakov et al., 2002) suggested they may be made more robust by considering networks that integrate in discrete bursts, rather than continuously. This can be implemented by considering a population of bistable neural units so that firing rate integration of a stimulus occurs in a stairstep fashion, rather than a ramplike fashion (see Goldman et al., 2003 for example). Related ideas were recently implemented in a bump attractor model of spatial working memory (Kilpatrick et al., 2013), but quantization was implemented with synaptic architecture rather than single neural unit properties. As opposed to the approach of quantizing the space of possible stimulus representations, we have kept the representation space a continuum. Deleterious effects of noise are reduced by considering reciprocal connectivity between encoding areas that redundantly represent the stimulus. Due to noise cancelations, the encoding error of the network decreases as the number of areas is increased.

### Conflict of interest statement

The author declares that the research was conducted in the absence of any commercial or financial relationships that could be construed as a potential conflict of interest.
